# CD8 and CD4 Positive NKT Subpopulations and Immune-Checkpoint Pathways in Early-Onset Preeclampsia and Healthy Pregnancy

**DOI:** 10.3390/ijms24021390

**Published:** 2023-01-10

**Authors:** Matyas Meggyes, Timoteus Feik, David U. Nagy, Beata Polgar, Laszlo Szereday

**Affiliations:** 1 Department of Medical Microbiology and Immunology, Medical School, University of Pecs, 12 Szigeti Street, 7624 Pecs, Hungary; 2 Janos Szentagothai Research Centre, 20 Ifjusag Street, 7624 Pecs, Hungary; 3 Institute of Geobotany/Plant Ecology, Martin-Luther-University, Große Steinstraße 79/80, 06108 Halle (Saale), Germany

**Keywords:** NKT, immune checkpoint, PD-1, LAG-3, TIGIT, preeclampsia

## Abstract

Although many studies have investigated the clinical aspect of early-onset preeclampsia, our knowledge about the immunological consequences of improper placenta development is scarce. The maternal immunotolerance against the fetus is greatly influenced by the Th1 predominance developed by the mother’s immune system. Thirty-two early-onset preeclamptic and fifty-one healthy pregnant women with appropriately matched gestational age were involved in our study. Mononuclear cells were separated from peripheral venous blood and the frequency of CD8⁺, CD4⁺, double positive (DP), and double negative (DN) NKT cell subpopulations was determined using multicolor flow cytometry. Following the characterization, the expression levels of different immune checkpoint receptors and ligands were also defined. Soluble CD226 levels were quantified by ELISA. Novel and significant differences were revealed among the ratios of the investigated NKT subsets and in the expression patterns of PD-1, LAG-3, TIGIT and CD226 receptors. Further differences were determined in the expression of CD112, PD-1, LAG-3 and CD226 MFI values between the early-onset preeclamptic and the healthy pregnant groups. Our results suggest that the investigated NKT subpopulations act differently in the altered immune condition characteristic of early-onset preeclampsia and indicate that the different subsets may contribute to the compensation or maintenance of Th1 predominance.

## 1. Introduction

Preeclampsia is a gestational hypertensive disorder affecting 5–8% of pregnancies [1]. This heterogeneous syndrome consists of endothelial and placental dysfunction, making preeclampsia a major cause of severe maternal and fetal morbidity and mortality [2]. It is characterized by increased blood pressure in previously normotensive women, accompanied by proteinuria after 20 weeks of gestation [1]. The disease is subdivided into early- and late-onset forms according to the time of occurrence [3]. Early-onset preeclampsia (EOP) develops after 20 weeks of gestation, while the late-onset form usually occurs after 34 weeks of pregnancy [4]. EOP is primarily linked to insufficient invasion of the extravillous cytotrophoblast and a maladaptation of the maternal spiral artery remodeling in the early phase of pregnancy, resulting in inadequate placentation and a small-sized placenta [5]. The clinical appearance of the symptoms usually occurs after 20 weeks of gestation when the growth of the fetus accelerates, and the abnormal placenta cannot compensate for it. The inadequate trophoblast invasion and poor artery remodeling finally lead to endothelial dysfunction and oxidative burst, which is linked to the appearance of inflammatory factors and antiangiogenic proteins (soluble fms-like tyrosine kinase 1, soluble endoglin) in the maternal circulation, and trigger a systemic Th1 type immunological environment [6].

Understanding the mechanisms behind the altered immunological processes in a preeclamptic patient is crucial since the Th1 shift could lead to the clinical manifestations of pregnancy complications and preterm birth. So far, only limited data are available about the subpopulations of natural killer T (NKT) cells and their potential role in this altered immune balance. NKT cells are a diverse population and part of the innate immune system considered as innate-like T cells [7,8]. Functionally, the NKT cells can be divided into two subpopulations (type I and type II) based on the differences regarding their T cell receptors [9,10]. Type I NKT cells or invariant NKT cells are less frequent and could recognize the glycolipid α-galactosylceramide, and their TCR is restricted to bind with CD1d [11].

In contrast, the TCR of the type II NKT subset is more diverse and has a broader antigen specificity. At the same time, it could also interact with CD1d [12]. The type II subset is more frequent and is comprised of CD4⁺, CD8⁺, and CD4⁻CD8⁻ (double-negative (DN)) T cells [8]. Previous studies published the regulatory roles of the type II NKT cells, suggesting their potential role in maintaining immunological homeostasis by suppressing anti-tumor immunity [13,14].

Immune checkpoint (IC) molecules mediate co-stimulatory and co-inhibitory signals and play a major role in maintaining the immunological balance between tolerance and autoimmunity. While co-stimulatory signals transmitted by activating receptors could enhance the proliferation and cytotoxicity of T cells, co-inhibitory signals mediated by inhibitory receptors could reduce the function of effector cells and induce apoptosis. During the last 20 years, numerous IC molecules have been discovered and evaluated as potential therapeutic targets, especially in cancer immunotherapy. 

Programmed death 1 (PD-1) and its ligand PD-L1 are the most studied IC molecules. Their interaction with immune cells and their use as an immune therapy are well documented [15,16,17]. PD-1 is primarily expressed by activated T cells and, following the interaction with PD-L1 or PD-L2 protein, it mediates an inhibitory signal to the receptor-positive cells, which helps to maintain the immunological balance between inflammation, immunity and tolerance [18]. The possible role of the inhibitory effect of PD-1/PD-L1 interaction by T cells in EOP was published in our previous work [19]. However, there are no scientific data about the significance of these checkpoint molecules expressed by different NKT subpopulations.

Lymphocyte-activation gene 3 (LAG-3/CD223) is an inhibitory receptor predominantly expressed by the activated T cells [20], which mediates inhibitory signals similarly to PD-1 or CTLA-4 [21]. Its surface expression by T cells is influenced by IL-2, IL-7 and IL-12A/B [22] and, after a ligation, LAG-3 inhibits the activation and proliferation of T cells [23]. Interestingly the structure of LAG-3 is close to the CD4 molecule; therefore, it can bind to the MHC-II, which negatively influences T cell activation and cytotoxicity and down-regulates the antigen response of helper T lymphocytes [24]. LAG-3 can bind to multiple ligand molecules such as fibrinogen-like protein 1 (FGL1), LSECtin or Galectin-3 (Gal-3). The interaction between LAG-3 and Gal-3 leads to the reduction of IFN-γ release in LAG-3 sufficient cells [25]. 

T cell immunoglobulin and ITIM domain (TIGIT) is a more recently published IC receptor primarily expressed by T- and NK cells that has an important role in immune regulation. Similar to PD-1, TIGIT mediates inhibitory signals after binding to one of its ligands, which can inhibit the cytotoxic activity of NK cells [26] and T cells [27]. CD226 is also a transmembrane receptor considered a co-stimulatory counterpart of TIGIT and is usually expressed by T-, NK cells and monocytes [28]. In contrast to TIGIT, the CD226 molecule transmits activating signals and can promote the effector function in the receptor-expressing cells [29]. These receptors can bind to the same ligands, either CD155 or CD112 [30]. These ligand molecules are usually expressed on the surface of the antigen-presenting cells (dendritic cells and monocytes). Still, their presence was also detected in non-hematopoietic tissues such as kidneys, intestines, and the nervous system [31,32,33]. In addition, a CD155 overexpression was also found in human malignant tumors [34]. Since both receptors can interact with the same ligands, TIGIT and CD226 compete for ligand binding, although TIGIT has a greater affinity with CD155 than with CD226 [27]. Furthermore, the TIGIT/CD155 ligation can directly inhibit the activatory signal transduction by destroying the homodimer of the CD226 molecule [35,36].

## 2. Results

### 2.1. The Frequency of NKT Cell Subpopulations in EOP Patients and Healthy Pregnant Women

**Figure 1:**
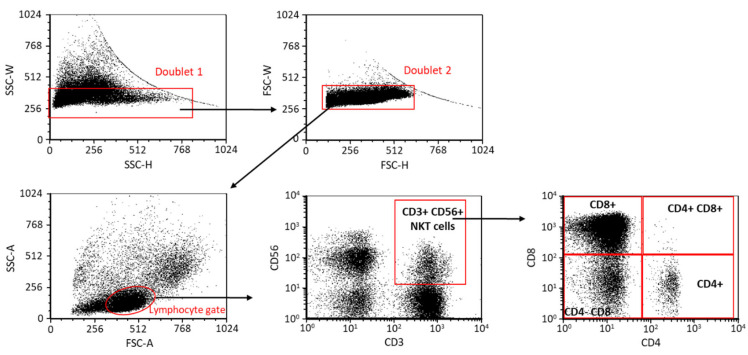
Differentiation of the NKT subpopulations using flow cytometric analyses. Gating strategy for flow cytometry analysis. The selection method to gate the four investigated NKT cell subpopulations.

**Figure 2:**
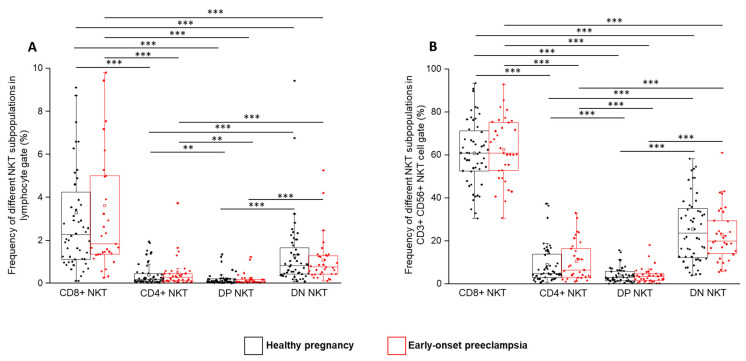
The frequency of different NKT subpopulations in EOP and healthy pregnancy. The frequency of the CD8⁺, CD4⁺, DP, DN NKT cells in the lymphogate (**A**) and the CD3⁺CD56⁺ NKT gate (**B**) in EOP patients and healthy pregnant women. The solid bars represent medians of 51 and 32 determinations, respectively. The boxes indicate the interquartile ranges, and the whiskers show the most extreme observations. The middle square within the box represents the mean value. Significant differences with *p*-values < 0.01 *** and <0.03 ** are indicated.

Using the flow cytometric gating strategy (Figure 1.), different NKT cell subpopulations (CD8⁺/CD4⁻ (CD8⁺), CD8⁻/CD4⁺ (CD4⁺), CD8⁺/CD4⁺ (DP), CD8⁻/CD4⁻ (DN)) were identified based on the presence of CD8 and CD4 surface markers. Notably, significant differences were revealed in the frequency of the examined subsets obtained from the peripheral blood of EOP patients and healthy pregnant women (Figure 2.). The major subpopulation is the CD8⁺/CD4⁻ subset, followed by the DN, the CD8⁻/CD4⁺ and the DP subsets. The frequencies of the NKT cell subpopulations both in the lymphogate (Figure 2A) and the CD3⁺CD56⁺ NKT gate (Figure 2B) significantly differ from each other within one tested cohort. However, there was no significant difference between the EOP patients and healthy pregnant women. 

### 2.2. PD-1 Receptor and PD-L1 Ligand Expression by the Four NKT Cell Subpopulations in EOP Patients and Healthy Pregnant Women

**Figure 3:**
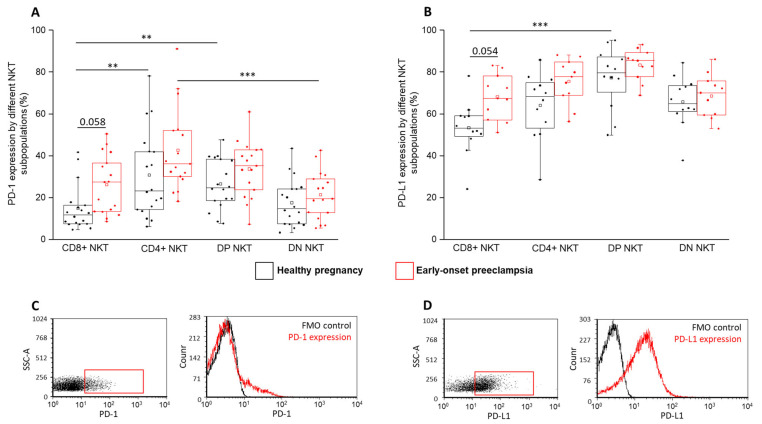
PD-1 and PD-L1 expression by different NKT cell subpopulations in EOP and healthy pregnancy. PD-1 receptor expression (**A**) and PD-L1 ligand expression (**B**) by the CD8 and CD4 positive and negative NKT cell subpopulations in EOP patients and healthy pregnant women. The solid bars represent medians of 18 and 17 determinations, respectively. The boxes indicate the interquartile ranges, and the whiskers show the most extreme observations. The middle square within the box represents the mean value. Significant differences with *p*-values < 0.01 *** and <0.03 ** are indicated. Representative FACS plots show the PD-1 surface marker (**C**) and PD-L1 surface molecule (**D**) expression by cells in the lymphocyte gate. Fluorescent minus one (FMO) control was used to determine PD-1 and PD-L1 expression.

The surface expression of PD-1 and PD-L1 molecules by NKT cells was measured by flow cytometry (Figure 3). We observed a significantly decreased PD-1 expression by the CD8⁺ subset compared to the CD4⁺ and DP subsets in a healthy pregnancy. Moreover, in EOP, significantly increased receptor expression was revealed by the CD4⁺ cell population compared to that of the DN subset (Figure 3A). Furthermore, an increasing tendency was also observed in the PD-1 expression by the preeclamptic CD8⁺ subset compared to the healthy counterpart (Figure 3A). A significantly elevated PD-L1 expression by the DP subset was measured in a healthy pregnancy compared to the CD8⁺ NKT cells (Figure 3B). Similar to the PD-1 receptor, the expression of the PD-L1 ligand by the preeclamptic CD8⁺ subset showed an increasing tendency compared to in a healthy condition (Figure 3B).

### 2.3. LAG-3 Receptor and Gal-3 Ligand Expression by the Four NKT Cell Subpopulations in EOP Patients and Healthy Pregnant Women

**Figure 4:**
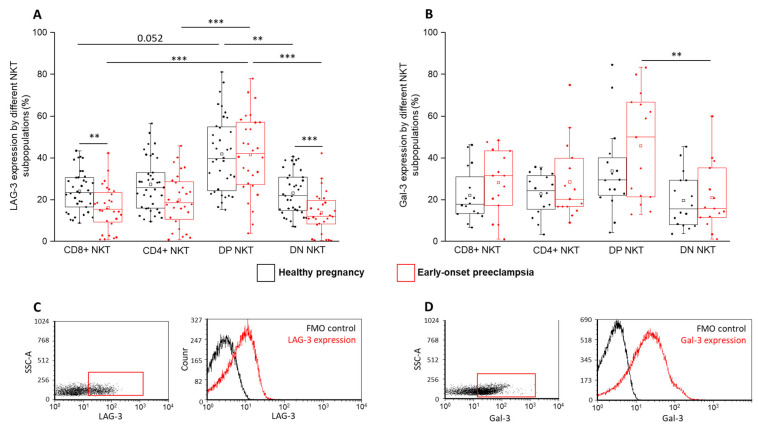
LAG-3 and Gal-3 expression by different NKT cell subpopulations in EOP and healthy pregnancy. LAG-3 receptor (**A**) and Gal-3 ligand expression (**B**) by the CD8 and CD4 surface molecule positive and negative NKT cell subpopulations in EOP patients and healthy pregnant women. The solid bars represent medians of 37, 28, 16 and 15 determinations, respectively. The boxes indicate the interquartile ranges, and the whiskers show the most extreme observations. The middle square within the box represents the mean value. Significant differences with *p*-values < 0.01 *** and <0.03 ** are indicated. Representative FACS plots show the expression of LAG-3 (**C**) and Gal-3 molecule (**D**) by cells in the lymphocyte gate. FMO control was used to determine LAG-3 and Gal-3 positivity.

Analyzing LAG-3, another inhibitory IC receptor, a significantly elevated surface expression by the DP subset was observed compared to the CD8⁺ and DN subpopulations in the EOP group (Figure 4A). This significant difference was also observed between the DP and CD4⁺ subsets in the EOP group. Comparing the LAG-3 surface expression between the two investigated cohorts revealed a significant decrease in the CD8⁺ and DN subsets in EOP compared to in healthy pregnant women (Figure 4A). In EOP patients, the intracellular Gal-3 level was significantly lower in the DN subpopulation compared to the DP cells (Figure 4B).

### 2.4. TIGIT and CD226 Receptor Expression by the Four NKT Cell Subpopulations in EOP Patients and Healthy Pregnant Women

**Figure 5:**
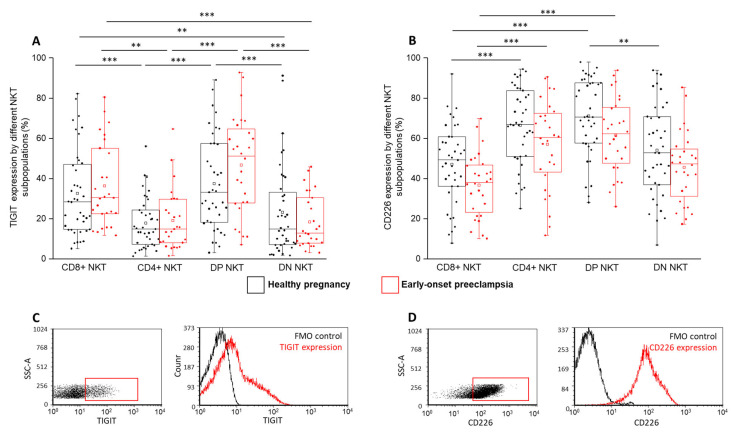
TIGIT and CD226 receptor expression by different NKT cell subpopulations in EOP and healthy pregnancy. TIGIT (**A**) and CD226 receptor expression (**B**) by the CD8 and CD4 surface molecule positive and negative NKT cell subpopulations in EOP patients and healthy pregnant women. The solid bars represent medians of 39, 40, 39 and 28 determinations, respectively. The boxes indicate the interquartile ranges, and the whiskers show the most extreme observations. The middle square within the box represents the mean value. Significant differences with *p*-values < 0.01 *** and <0.03 ** are indicated. Representative FACS plots show the TIGIT (**C**) and CD226 surface molecule (**D**) expression by cells in the lymphocyte gate. FMO control was used to determine TIGIT and CD226 expression.

During flow cytometric measurements, notable differences were observed regarding the expression of TIGIT in both cohorts. A significantly increased inhibitory receptor expression was measured by the CD8⁺ and DP subsets compared to the CD4⁺ and DN subsets (Figure 5A). No significant difference was observed between the EOP patients and healthy pregnant women. Investigating the surface expression of the activatory CD226 receptor, a significantly elevated presence was found in the CD4⁺ and DP subsets compared to the CD8⁺ subset in both studied groups (Figure 5B). Furthermore, the CD226 expression was significantly increased by the DP subset compared to the DN subset in a healthy pregnancy (Figure 5B). A significant difference between the EOP patients and healthy women was not detected.

### 2.5. CD112 and CD155 Ligand Expression by the Four NKT Cell Subpopulations in EOP Patients and Healthy Pregnant Women

**Figure 6:**
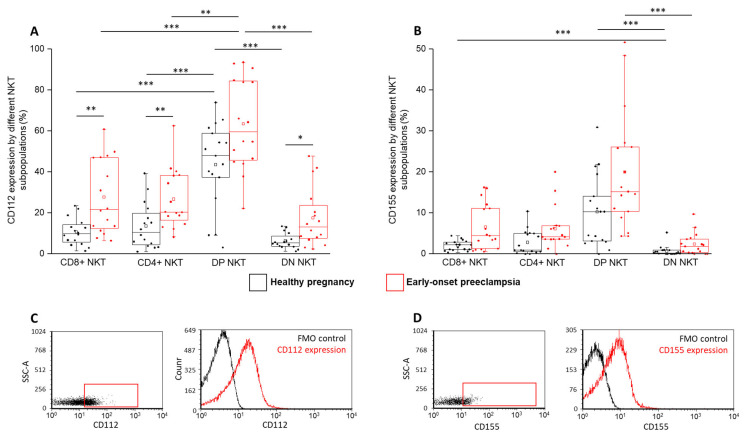
CD112 and CD155 ligand expression by different NKT cell subpopulations in EOP and healthy pregnancy. CD112 (**A**) and CD155 ligand expression (**B**) by the CD8 and CD4 surface molecule positive and negative NKT cell subpopulations in EOP patients and healthy pregnant women. The solid bars represent medians of 16, 16, 17 and 17 determinations, respectively. The boxes indicate the interquartile ranges, and the whiskers show the most extreme observations. The middle square within the box represents the mean value. Significant differences with *p*-values < 0.01 ***, <0.03 ** and <0.05 * are indicated. Representative FACS plots show the CD112 surface marker (**C**) and CD155 surface molecule (**D**) expression by cells in the lymphocyte gate. FMO control was used to determine CD112 and CD155 expression.

After TIGIT and CD226 receptor analyses, we measured the expression of the IC ligand molecules on the surface of the investigated NKT subsets. A significantly increased CD112 expression was observed by the DP subsets compared to the CD8⁺, CD4⁺ and DN subpopulations in both healthy and EOP cohorts (Figure 6A). After further comparison, a significantly decreased CD112 expression was observed by the CD8⁺, CD4⁺ and DN subsets in healthy pregnant women compared to patients diagnosed with EOP (Figure 6A). In the case of CD155, a significantly decreased surface expression was revealed by the DN subset compared to the DP cells in both investigated groups (Figure 6B). Furthermore, this difference was also detected between the DN and CD8⁺ subsets in healthy conditions (Figure 6B).

### 2.6. Relative TIGIT and CD226 Expression by the Four NKT Cell Subpopulations in EOP Patients and Healthy Pregnant Women

**Figure 7:**
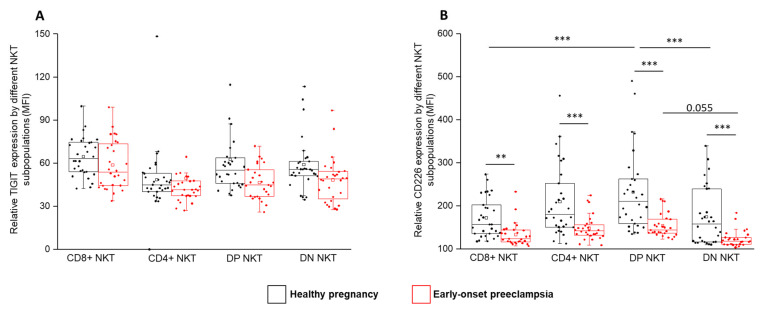
Relative TIGIT and CD226 expression by different NKT cell subpopulations in EOP and healthy pregnancy. Mean fluorescent intensity of the TIGIT (**A**) and the CD226 (**B**) receptors by the CD8 and CD4 surface molecule positive and negative NKT cell subpopulations in EOP patients and healthy pregnant women. The solid bars represent medians of 39, 40, 39 and 28 determinations, respectively. The boxes indicate the interquartile ranges, and the whiskers show the most extreme observations. The middle square within the box represents the mean value. Significant differences with *p*-values < 0.01 *** and <0.03 ** are indicated.

After the surface analyses, we examined the mean fluorescent intensity (MFI) of inhibitory TIGIT and activatory CD226 receptors. Although a significant difference has not been detected regarding the MFI of TIGIT expression, the relative expression of CD226 was significantly lower in EOP patients compared to healthy controls in all four NKT subpopulations. Furthermore, in healthy patients, a significantly elevated CD226 MFI was determined in the DP subset compared to the CD8⁺ and DN subsets (Figure 7B).

### 2.7. The Serum Level of CD226 and the Relationship between the Surface and the Relative Expression of CD226 by NKT Cell Subsets in EOP Patients and Healthy Pregnant Women

**Figure 8:**
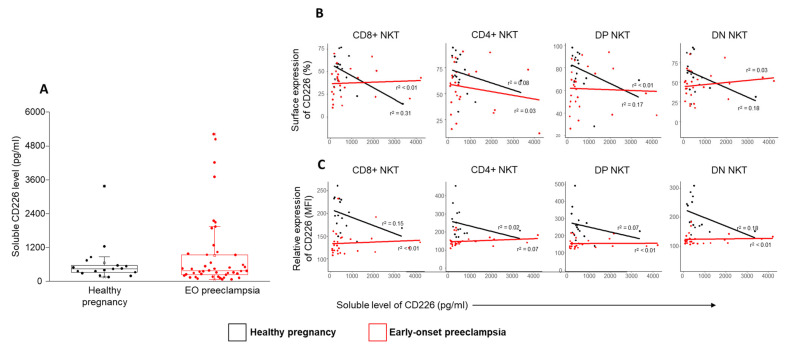
Relationship between the surface expression or the relative expression of CD226 and the level of soluble CD226 in NKT subpopulations in EOP and healthy pregnancy. The serum concentration of CD226 (**A**) molecule in EOP patients and healthy control women. The solid bars represent medians, the boxes indicate the interquartile ranges and the lines show the most extreme observations. Linear regression analyses between the CD226 surface (**B**) or relative expression (**C**) with the sCD226 level in different NKT subsets in EOP and healthy control women. *p* values and coefficients of determination (R^2^) were calculated in R.

Analyzing the serum level of the soluble CD226 molecule, a statistically significant difference was not detected between the investigated groups (Figure 8A.). Additionally, no correlation was found between the surface or relative expression of CD226 by different NKT subsets and the serum CD226 (sCD226) levels (Figure 8B).

## 3. Discussion

The clinical aspect of EOP is well examined [37,38,39], but much fewer data are available about the immunological background of the disease. Besides the systemic Th1 immune response in preeclamptic women, Valencia-Ortega et al. published a possible connection between the imbalance of pro- and anti-inflammatory cytokine release and endothelial dysfunction [40]. Further studies reported a reduced ratio of peripheral regulatory T cells (Treg) and Th17 subpopulations in preeclampsia [41,42]. Other papers revealed a decreased number of decidual Treg and decidual NK cells in preeclamptic patients compared to healthy pregnant individuals [43,44]. Since NKT cells share morphological and functional characteristics with both NK and T cells, they work as a bridge between innate and adaptive immunity [45]. However, their potential role during the pathogenesis of EOP is still not well studied. Recent publications emphasize the significance of type II NKT cells in maintaining immunological homeostasis; therefore, investigating IC molecules in an altered immunological environment such as EOP might help us better understand the disease pathology.

Examining the best-known IC pathway, the lowest PD-1 and PD-L1 expression was measured in the CD8⁺ NKT subset, the largest subset of the four. These differences were detected only in a healthy pregnancy, which refers to an altered immunological environment in EOP. Furthermore, we hypothesize that the increased tendency of the PD-1 and PD-L1 expression by the CD8⁺ subset in EOP patients might be a part of a compensatory mechanism against the Th1 predominance. In connection with this, some researchers published an elevated PD-1 expression and consequent functional exhaustion of the NKT cell population, which was restored after an anti-PD-1 treatment in cancer patients [46,47]. Since activated NKT cells can produce inflammatory cytokines (e.g., IFN-γ, TNF and IL-2) [48,49], the investigated CD8⁺ NKT cells in EOP patients might be functionally inactive to diminish the harmful effect of the Th1 immune environment. 

Investigating the expression of LAG-3, an opposite pattern was detected compared to the PD-1 results, since the presence of the LAG-3 receptor was reduced only by the CD8⁺ and DP subsets in EOP patients. Therefore, the lack of the CD4 marker in the CD8⁺ NKT cells and the decreased LAG-3 surface expression in EOP may reflect their potential cytotoxic feature. Recent human and mice studies investigating tumor immunology emphasize the importance of the LAG-3 molecule in PD-1 immunotherapy [21], since the simultaneous inhibition of LAG-3 and PD-1 increases the antitumor immunity of antigen-specific T cells compared to the single blockade either by PD-1 or by LAG-3 alone [21,50]. Based on these results, PD-1 and LAG-3 may function in synergy in a tumor microenvironment. At the same time, our investigations focusing on these inhibitory receptors, especially expressed by the major CD8⁺ NKT subsets, suggest a strong influence of the systemic inflammatory immune response on these IC pathways in EOP.

Aware of the above results, we considered it worthwhile to examine the TIGIT/CD226/CD112/CD155 IC network in the four NKT subsets. In the case of TIGIT, a reduced expression was detected by the CD4⁺ and DN subsets. CD8⁺ NKT cells expressed the inhibitory receptor at a higher level. On the contrary, the activatory receptor expression was decreased by the CD8⁺ and DN subsets, which means that CD4⁺ NKT cells expressed the activatory receptor at an increased level. However, the receptor expression levels did not differ in the investigated groups. Our results suggest that the CD4⁺ NKT cells are potentially more active than the CD8⁺ NKT subset, which may indicate the priority of the regulatory function of the NKT cells besides cytotoxicity. Interestingly, a reduced CD226 MFI was measured in EOP patients in all the investigated subsets. Takahashi et al. published that the soluble CD226 molecule could be shed from the membrane of NK cells and CD8⁺ T cells [51]. Since the MFI value correlated with the amount of the surface molecule, we hypothesized that a reduced CD226 level might be found in the circulation. We did not detect any significant difference between the sCD226 levels or during the regression analyses between the surface or the relative expression of CD226. It is possible that the CD226 molecule shed from the surface of NKT cells does not stay too long in circulation but interacts with the increased amount of CD112 and CD155 ligands expressed by the monocytes, NK and T cell subsets [52]. This interaction can block these ligands from connecting with surface TIGIT or CD226 receptors. According to this hypothesis, the physiological function of the TIGIT/CD226/CD112/CD155 IC network is damaged and might contribute to the altered immune environment characteristic of EOP.

Since the IC pathways are vital regulatory molecules for maintaining self-tolerance and immunological homeostasis, based on our results, we assume that the investigated IC pathways have significant roles in the modification of maternal immunotolerance in EOP. Although our data do not clearly define the potential impact of the different NKT cell subpopulations, the revealed differences in the expression pattern of these novel IC molecules by the four NKT subsets indicate their involvement in the systemic Th1 predominance seen during EOP. Furthermore, the revealed differences in the expression level of the PD-1/PD-L1 and CD226/CD112 IC interactions in EOP might refer to their clinical relevance and could be a basis for future immune therapies. Although no significant difference was found in the amount of the soluble CD226, its follow-up from the first trimester might have diagnostic value and could be an indicator of future EOP.

## 4. Materials and Methods

### 4.1. Ethical Approval

Informed, written consent was obtained from all participants in accordance with a protocol approved by the Regional and Local Research Ethics Committee at the Medical School, University of Pecs, Hungary (Reference number: 6149). The study protocol conforms to the ethical guidelines of the 1975 Declaration of Helsinki. 

### 4.2. Participants and Sample Collection

Thirty-two pregnant women diagnosed at the Department of Obstetrics and Gynecology, University of Pecs, with the classic symptoms of preeclampsia (hypertension, proteinuria) were involved in this case-control study (Table 1). The diagnosis of early-onset preeclampsia was established according to the ISSHP definition: increased blood pressure (≥140 mmHg systolic or ≥90 mmHg diastolic on ≥2 separate occasions at least 4 h apart within a 24 h period) that occurred before the 34th week of gestation in women with previously normal blood pressure, accompanied by organ failure, such as significant proteinuria (≥30 mg/mol protein in 24-h urine collection in the absence of urinary tract infection). Then, 20 mL of heparinized venous blood was collected and transported immediately to the laboratory.

Fifty-one healthy pregnant volunteers enrolled at the Department of Obstetrics and Gynecology, University of Pecs, with appropriately matched gestational age, served (Table 1) as a control group. Under the European Union General Data Protection Regulation (GDPR) and Protection of Health and Related Personal Data Act Regulations, all personal and health-related data obtained during blood donation were processed anonymously, confidentially and securely. The health status of each of the control women was evaluated. None of them had a significant medical history, were taking medications, including hormonal contraceptives, nor had any current illnesses. All women affected by pregnancy-related complications and/or infection, pre-pregnancy disease, in vitro fertilization pregnancies, immune-associated disease, diabetes mellitus or AIDS were excluded. None of the participants were tobacco consumers/smokers.

### 4.3. Lymphocyte Separation, Cryopreservation, and Thawing

Peripheral blood mononuclear cells (PBMCs) were isolated from heparinized venous blood using Ficoll Paque (GE-Healthcare, Chicago, IL, USA) density gradient. The PBMC fraction was washed in complete Rosewell Park Memorial Institute medium 1640 (RPMI, Lonza, Switzerland) supplemented with 10% fetal calf serum (FCS, Lonza, Basel, Switzerland), then counted and centrifuged. Next, the pelleted cells were resuspended in inactivated human AB serum containing 10% DMSO for cryoprotection (Sigma-Aldrich, St. Louis, MO, USA), then aliquoted in 1 mL cryovials and stored in a −80 °C mechanical freezer for further flow cytometric investigation. On the day of the experiment, the samples were thawed in a 37 °C water bath, resuspended in RPMI 1640 medium, and washed twice to remove the remaining DMSO content.

### 4.4. Flow Cytometric Measurement

Briefly, fluorochrome-conjugated monoclonal antibodies (Table 2) were added to 106 PBMCs and incubated for 30 min at room temperature (RT) in darkness. After a washing step, cells were resuspended in 300 µL phosphate-buffered saline (PBS) (BioSera, Nuaillé, France) containing 1% paraformaldehyde (PFA) and stored at 4 °C in complete darkness until flow cytometric analysis. Flow cytometric measurements were performed using a BD FACS Canto II flow cytometer (BD Immunocytometry Systems, Erembodegem, Belgium) with the BD FACS Diva V6. Software (BD Biosciences, San Jose, CA, USA) for data acquisition. Flow cytometric data were performed by FCS Express V4 (De Novo Software, Pasadena, CA, USA).

### 4.5. Intracellular Staining 

Following surface labeling, cells were washed with PBS and fixed in 4% PFA for 10 min at RT in darkness. After the cells were washed with PBS, they were permeabilized with 1:10 diluted FACS Permeabilizing Solution 2 (BD Biosciences) for 10 min at RT in darkness. Then the samples were washed and incubated with PE-conjugated anti-human galectin-3 for 30 min at RT in darkness. The samples were washed with PBS, fixed with 1% PFA, and stored at 4 °C in darkness until FACS analysis.

### 4.6. Enzyme-Linked Immunosorbent Assay (ELISA)

Serum Nectin-2 (CD112) and DNAM1 (CD226) concentrations were determined by sandwich Enzyme-Linked Immunosorbent Assays (ELISA) according to the previously published methods [53]. Briefly: (1) Nectin-2/CD112 ELISA (InvitrogenTM, Thermo Fisher Scientific, Waltham, MA, USA, EH331RB): 100 µL of diluted standards and 2-fold diluted sera were pipetted to a test-plate pre-coated with anti-human Nectin-2 antibody and were incubated for 2.5 h at RT. After washing four times, 100 µL of biotin-conjugate was added to the wells and incubated for 45 min at RT. After washing, the enzyme reaction was developed with 100 μL/well of TMB substrate for 30 min at RT in darkness and terminated with a stop solution; (2) In the case of DNAM-1/CD226 ELISA (ELISA Kit: R&D Systems, Bio-Techne, Minneapolis, MN, USA, DY666-05, Ancillary Reagent kit 2: R&D Systems, Bio-Techne, DY008), a 96-well microplate was coated overnight with 100 µL/well of anti-human CD226 antibody at RT. Then, the plate was washed and blocked with 400 µL/well of Reagent diluent for an hour. After washing, 100 μL of serial-diluted standards and serum samples were added, and the plate was incubated for 2 h at RT. Then, the wells were washed, and the plate was incubated with 100 µL of diluted detection antibody for 2 h at RT. After another washing, the wells were incubated with 100 µL/well streptavidin-HRP for 20 min. After washing, the plate was developed with Substrate Reagents A + B (1:1) for 20 min in darkness. Finally, the reaction was terminated with a stop solution. The absorbance of the test plates was measured (1) at 450 nm or (2) at 450 nm with a reference filter of 540 nm using a BMG SPECTOstar Nano spectrophotometer (BMG Labtech, Ortenberg, Germany). Standard curves were generated after background subtraction by the 4-parametric logistic analysis, then the Nectin-2 and DNAM-1 levels were determined with MARS Data Analysis Software version 3.32 (BMG Labtech, Ortenberg, Germany). In the case of CD112-ELISA, the calculated concentrations were corrected by a dilution factor of two.

### 4.7. Data Analysis

To analyze the differences between the investigated parameters of NKT subpopulations (CD4⁺, CD8⁺, DP and DN) and patients’ demographic and gynecological characteristics, linear models were run in R. Decisions on the transformation of response variables depended on a visual inspection of “model-checking plots” for the models with transformed vs. untransformed variables. Based on these plots, the normality of residuals and the assumption of the homogeneity of variance were checked [54]. Variables were log-transformed. Each response variable (expression patterns of PD1, TIGIT, etc.) was analyzed separately in the models. Explanatory variables were the two-way interaction effects of cell types × patient health status. A two-way ANOVA was used to test for statistical significance. For pair-wise comparisons, Tukey post-hoc tests were conducted with the multcomp-package [55] to compare each cell type/status combination to each other.

## Figures and Tables

**Table 1:** Patients’ demographic and gynecological characteristics.

	Healthy Pregnant Women	Early-Onset Preeclamptic Patients
No. of patients	51	32
Age (years)	32.84 (23–44)	29.81 (18–43)
Gestational age at birth (weeks)	39.06 ± 0.97	31.31 ± 3.17 *
Gestational age at sampling (weeks)	33.11 ± 3.91	30.36 ± 2.53
Birth weight (gram)	3447.38 ± 411.04	1429.14 ± 599.97 *

Statistical comparisons were made using the independent sample *t*-tests. The results are expressed as the mean value ± standard deviation of the mean. * *p* < 0.01 vs. healthy pregnant women.

**Table 2:** Fluorochrome-conjugated monoclonal antibodies used in the study.

Antigen	Format	Clone	Isotype	Company	CAT
CD112	PE	R2.525	Mouse IgG1, κ	BD Biosciences	551057
CD155	APC	SKII.4	Mouse IgG1, κ	Biolegend	337618
CD3	BV510	UCHT1	Mouse BALB/c IgG1, κ	BD Biosciences	563109
CD4	FITC	RPA-T4	Mouse IgG1, κ	BD Biosciences	555346
CD8	APC-H7	SK1	Mouse BALB/c IgG1, κ	BD Biosciences	560179
CD56	APC	B159	Mouse IgG1, κ	BD Biosciences	555518
CD226	BV421	DX11	Mouse BALB/c IgG1, κ	BD Biosciences	742493
Galectin-3	PE	B2C10	Mouse BALB/c IgG1, κ	BD Biosciences	565676
LAG-3	PerCp Cy5.5	11C3C65	Mouse IgG1, κ	Biolegend	369312
TIGIT	PE	A1553G	Mouse IgG2a, κ	Biolegend	372704

## Data Availability

The data presented in this study are available on request from the corresponding author.
